# Chemopreventive Effects of Germinated Rough Rice Crude Extract in Inhibiting Azoxymethane-Induced Aberrant Crypt Foci Formation in* Sprague-Dawley* Rats

**DOI:** 10.1155/2017/9517287

**Published:** 2017-01-02

**Authors:** Elnaz Saki, Latifah Saiful Yazan, Razana Mohd Ali, Zalinah Ahmad

**Affiliations:** ^1^Laboratory of Vaccines and Immunotherapeutics, Institutes of Bioscience, Universiti Putra Malaysia (UPM), 43400 Serdang, Selangor, Malaysia; ^2^Department of Biomedical Science, Faculty of Medicine and Health Sciences, Universiti Putra Malaysia (UPM), 43400 Serdang, Selangor, Malaysia; ^3^Department of Pathology, Faculty of Medicine and Health Sciences, Universiti Putra Malaysia (UPM), 43400 Serdang, Selangor, Malaysia

## Abstract

Chemoprevention has become an important area in cancer research due to low success rate of current therapeutic modalities. Diet plays a vital role in the etiology of cancer. This research was carried out to study the chemopreventive properties of germinated rough rice (GRR) crude extract in* Sprague-Dawley* rats induced with azoxymethane. Germination of rough rice causes significant changes in several chemical compositions of presently bioactive compounds. These compounds may prevent or postpone the inception of cancer. Fifty male* Sprague-Dawley* rats (6 weeks of age) were randomly divided into 5 groups which were (G1) induced with azoxymethane (AOM) and not given GRR (positive control), (G2) induced with AOM and given 2000 mg/kg GRR, (G3) induced with AOM and given 1000 mg/kg GRR, (G4) induced with AOM and given 500 mg/kg GRR, and (G5) not induced with AOM and not given GRR crude extract (negative control). To induce colon cancer, rats received two IP injections of AOM in saline (15 mg/kg) for two subsequent weeks. Organs were removed and weighed. Aberrant crypt foci (ACF) were evaluated histopathologically. *β*-Catenin expressions were determined by Western blot. Treatment with 2000 mg/kg GRR crude extract not only resulted in the greatest reduction in the size and number of ACF but also displayed the highest percentage of nondysplastic ACF. Treatment with 2000 mg/kg GRR also gave the lowest level of expression in *β*-catenin. Thus, GRR could be a promising dietary supplement for prevention of CRC.

## 1. Introduction

Colorectal cancer (CRC) is a cancer that originates in the colon or the rectum. It can also be known separately as colon or rectal cancer, based on the origin. The rate of CRC is increasing worldwide yearly [1]. It is the second highest cancer in frequency and one of the most common causes of death, affecting both men and women, worldwide. According to the American Cancer Society, there were 102,480 new cases of CRC and 40,340 new cases of rectal cancer in the US alone in 2014 [2].

CRC treatment consists of either single sense modality or a mixture of two or more of these treatments: surgery, radiation therapy, chemotherapy, and immunotherapy. Cancer patients receiving treatments often experience unpleasant side effects such as body pain, hair and appetite loss, diarrhoea, fatigue, constipation, blood disorders, and nervous system effects that compromise the quality of their life [3, 4].

Most of the natural products used in traditional remedy have promising scientific evidence with regard to their biological activities [5]. However, there is little information or evidence available concerning the possible toxicity that medicinal plants may cause to the consumers [6]. In relation to drug discovery and development, there are different weights of concern from the relevant groups such as health authorities, pharmaceutical industry, and patients which need to be taken into consideration [7]. The data derived from the acute and subacute toxicity studies on medicinal plants or preparations should be obtained in order to ensure safety to consumers and increase the confidence of the stakeholder, particularly when they are potentially ready for use in the development of pharmaceuticals [8].

Rice* (Oryza sativa)* is the staple diet of more than half the world's population, primarily in Asian countries where the occurrence of CRC is significantly lower than in Western countries [9, 10]. Previous findings showed that rice extract or the individual compound such as phytic acid of rice gave significant reduction in the formation of ACF [11–13].

Germinated rough rice (GRR) is rough rice, which has been soaked in water for up to a day and has a germ approximately 2 mm long. During germination, the chemical composition and active compounds level are increased significantly, resulting in the increase of simple sugars, peptides, and amino acids level in the germinated seeds. Other than modifying the level of nutrients, the biochemical activities that take place during the germination process can also release bioactive components, some of which contain antioxidants such as ascorbic acid, tocopherols, tocotrienols, and phenolic compounds, therefore resulting in an increase of antioxidant activity [14, 15].

Dimethylhydrazine and metabolic derivatives classically, 1,2-dimethylhydrazine (DMH) or its metabolite azoxymethane (AOM), have been used for inducing CRC in mice and rats. The AOM-induced colon carcinogenesis is an established model that has been used to evaluate the effectiveness of any preventive treatment [16]. Adenomatous polyps and carcinomas of the colon induced by AOM in rats were similar to those identified in humans [17]. Changes or irregularities in converting growth factor beta (TGF-*β*) signaling are found in tumors developed by mice induced with AOM. Induction with AOM activates intrinsic tyrosine kinase of EGF receptor and stimulates the synthesis of TGF-alpha. The cyclooxygenase-2 (COX-2) inhibitor NS-398 minimizes the occurrence of preneoplastic cells in AOM-induced rats [18, 19].

DMH- or AOM-induced tumors have many common histopathological characteristics of human CRC, and they frequently carry mutations in K-Ras and *β*-catenin. In human lesions, genes are often mutated or removed. K-ras and adenomatous polyposis coli (APC) gene mutations are elaborate in relative early steps of colon carcinogenesis, though alterations of deleted colorectal carcinoma (DCC) and p53 are involved in the late steps. They show microsatellite instability indicating a defective mismatch repair (MMR) system. During initiation of colon carcinogenesis, mutation in the APC gene is often one of the earliest events. APC mutations, however, are less frequent in rodents; p53 mutations are rarely observed; and the tendency to metastasize is low as has been summarized by Páez et al. [18, 20].

The objective of this study was to determine the chemopreventive properties of GRR crude extract in* Sprague-Dawley* rats induced with azoxymethane (AOM). AOM is a potent carcinogen used to induce colon cancer in rodents. It has been commonly used in studies evaluating efficacy of preventative treatment for azoxymethane-induced carcinogenesis.

## 2. Materials and Methods

Malaysian local rough rice* (Oryza sativa)* was obtained from a major rice miller of Padiberas Nasional Berhad (BERNAS) in Selangor, Malaysia.

### 2.1. Preparation of Germinated Rough Rice Powder

GRR was prepared according to the methods mentioned by Saetung [21] with slight modifications. Briefly, rough rice was soaked in distilled water (changed every 8 hours) for 48 hours at room temperature (28°C). The rice seeds were covered with wet napkin after soaking until germination occurred. The germinated seeds were dried at 50°C using an oven, to about 10% of moisture content. The hull, root, and shoot were separated to obtain GRR. Dried GRR was finely ground (40 mesh) into powder.

### 2.2. Preparation of Germinated Rough Rice Crude Extract

GRR powder was mixed with 95% ethanol (1 GRR : 3 ethanol (w/v)) and incubated at 50°C using an incubator shaker for 6 hours. The slurry was cooled, homogenized, and filtered through Whatman filter paper No. 2. The extracted slurry was subjected to rotary evaporation to remove the solvent and then freeze-dried to obtain germinated rice crude extract powder (GRP). GRP was stored at −80°C for further analysis.

### 2.3. Determination of Cytotoxicity of Germinated Rough Rice Crude Extract

Cytotoxicity of GRR crude extract on human colon cancer cells (HT-29) was determined by the MTT (3-(4,5-dimethylthiazol-2-Yl)-2,5-diphenyltetrazo-lium bromide) assay [22]. HT-29 cell line was obtained from the American Type and Culture Collection (ATCC, Rockville, MD). Briefly, 1 × 105 of cells were seeded in each well of a 96-well plate. After 24-hour incubation, the cells were treated with GRR crude extract (3.13 to 100 *μ*g/mL) and with the inclusion of untreated control cells. After incubation with GRR crude extract for 24, 48, and 72 hours, 20 *μ*L of 5 mg/mL of MTT was added into each well and incubated for 3 hours. Active mitochondria in live cells reduced MTT to crystalline purple blue formazan. The quantity of living cells was proportionate to the amount of crystalline purple blue formazan produced. After incubation, media in each well was discarded and 100 *μ*L of DMSO was added to solubilize the purple blue formazan. The absorbance was measured with an ELISA microplate reader (Biotek ELX 800 TC models 96 well, USA) at wavelength of 570 nm and 630 nm as background. A graph of percentage of cell viability versus concentration of GRR crude extract was plotted, and the IC50 (concentration that inhibits 50% of cell growth compared to control) was determined.

### 2.4. Ethical Approval

Ethical approval for the animal conduct was obtained from the Animal Care and Use Committee (ACUC), Faculty of Medicine and Health Sciences, Universiti Putra Malaysia (UPM/IACUC/AUP-R051/2013).

### 2.5. Determination of Chemopreventive Properties of Germinated Rough Rice Crude Extract

Five-week-old male* Sprague-Dawley* rats weighing 100–150 g were purchased from the Faculty of Medicine and Health Science, UPM. For the duration of the study period, the animals were housed in plastic cages with woodchip bedding in a ventilated room placed in a controlled environment with a 12 h light/dark cycle at 25 ± 2°C and 40–70% humidity. Woodchip bedding was regularly changed to maintain a hygienic condition. Each animal was examined for clinical signs of ill health on receipt and observed within 7 days of arrival. Body weight and food and water consumption were monitored daily. Animals were fasted approximately 12 hours before dosing.

### 2.6. Induction of Colon Cancer and Treatment

Briefly, fifty male* Sprague-Dawley* rats (6 weeks old) were randomly divided into 5 groups (*n* = 10). G1 was induced with AOM and unfed with GRR (positive control), G2 was induced with AOM and fed with 2000 mg GRR crude extract/kg body weight, G3 was induced with AOM and fed with 1000 mg GRR crude extract/kg body weight, G4 was induced with AOM and fed with 500 mg GRR crude extract/kg body weight, and G5 was not induced with AOM and unfed with GRR crude extract (negative control). Prior to treatment, the rats were initially injected intraperitoneally (IP) with AOM in saline (15 mg/kg body weight) over a 2-week (one time per week) period to induce colon cancer. GRR crude extract was administered orally once daily for 8 weeks via gavage. After 8 weeks of treatment, the animals were sacrificed by decapitation under chloroform anesthesia. Blood was collected via cardiac puncture and placed in tubes containing anticoagulant for hematological examinations. Colons and all the organs (liver, kidney, lung, heart, spleen, and colon) were removed, weighed, and fixed in 10% formalin.

### 2.7. Determination of Incidence of ACF

Colons were removed, cut along the longitudinal axis, and washed with phosphate buffered saline (PBS). Each colon was cut into three parts (proximal, middle, and distal) of equal length and fixed flat between filter papers in 10% buffered formalin (Mallinckrodt Specialty Chemicals Co., Paris, KY, USA) for at least 24 hours and stained with hematoxylin and eosin, before being subjected to standard histopathological analysis.

The presence of colonic aberrant crypt foci (ACF) was evaluated histopathologically. ACF were examined under 20x magnification using a light microscope (Nikon Corp., Tokyo, Japan) and distinguished from the normal crypts by their increased size, irregular and dilated luminal opening and thicker epithelial lining, and pericryptal zone. The number of ACF per colon, the number of aberrant crypts observed in each focus, and the location of each focus were all recorded.

### 2.8. Determination of the Effect of Germinated Rough Rice Crude Extract on Expression of *β*-Catenin

Colon tissue specimens were cut into 2-3 cm pieces and homogenized in lysis buffer with a homogenizer (IKA T18 Ultra-Turrax, Germany). The homogenate was centrifuged at 14,000 ×g for 10 minutes, and the supernatant was transferred into a new microcentrifuge tube. Next, the supernatant was again centrifuged at 14,000 ×g for 10 minutes to pellet the tissue debris. The clarified supernatant was then collected and stored at −80°C. The protein concentration quantification was performed by using Bradford Protein assay [23].

An equal amount of 10–20 *μ*g of proteins was separated by 10% SDS-PAGE. After electrophoresis, the proteins were transferred to PVDF membrane by semidry transfer method, blocked with 3% BSA in 0.1% Tween-20 containing Tris-Buffer Saline (TBS-T) at room temperature (20–25°C) for 1 hour, and reacted with anti-*β*-catenin (1 : 10,000), primary antibodies in TBS-T overnight at 4°C. After washing three times with TBS-T at room temperature, the primary antibodies were either reacted with horseradish peroxidase-conjugated goat anti-rabbit (1 : 40,000) secondary antibodies in TBS-T for 1 hour at room temperature. The protein visualization was then performed by using ChemiDoc MP System (Bio-Rad, Hercules, CA, USA) in a dark room and quantified by a densitometer analyzed using Bio-Rad Molecular Analyst software.

### 2.9. Statistical Analysis

Data analysis was carried out using Package for Social Science (SPSS) Version 20. Comparison of the difference between ACF and AC incidence among rats fed with GRR crude extract was made by One-Way ANOVA. The difference in body weight and organs was analyzed by Two-Way ANOVA. For multiple comparisons to realize the significance among each group and control group, Tukey's test was used. Calculation of the mean score was done by summing up the extent of five fields, selected at random from each section of colon/rat. Data were expressed as mean ± standard deviation (mean ± SD). A difference was considered to be significant at *p* < 0.05.

## 3. Results

### 3.1. Cytotoxicity of Germinated Rough Rice Crude Extract

**Figure 1:**
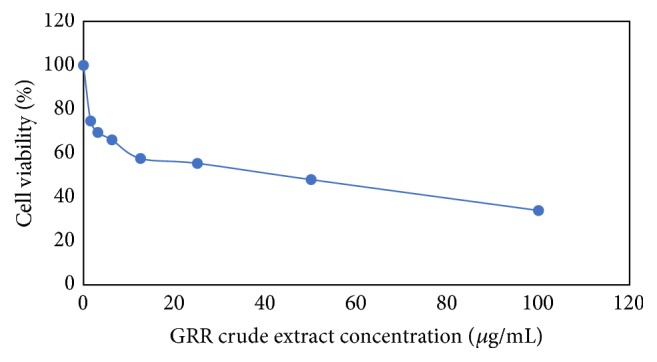
Effect of GRR crude extract on HT29 cells viability after 72 hours as determined by MTT assay.

The cytotoxic effect of GRR crude extract was time- and dose-dependent. IC50 value of GRR crude extract was 43 *μ*g/mL after 72 hours (Figure 1).

### 3.2. Chemopreventive Properties of Germinated Rough Rice Crude Extract in Rats Induced with Azoxymethane

#### 3.2.1. Body and Organs Weight

**Figure 2:**
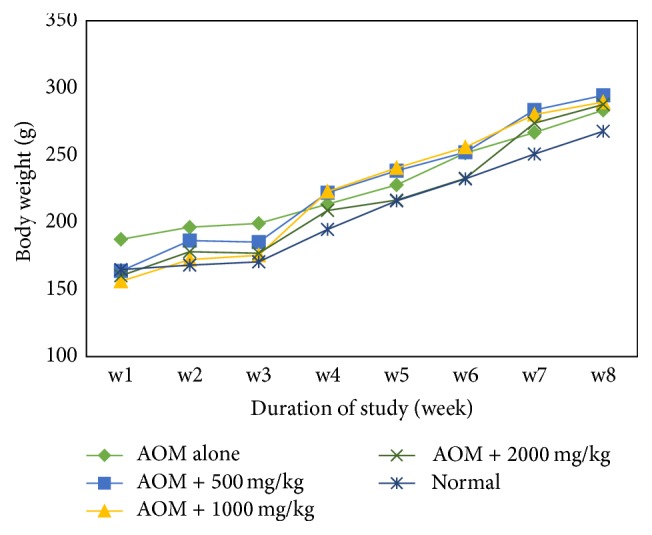
Body weight of rats induced with azoxymethane treated with different dose of germinated rough rice crude extract.

All animals treated and untreated with GRR crude extract survived during the entire course of the experiment.  Figure 2 shows the body weight of the rats treated with GRR crude extract and the control group. The body weight of the animals in all the groups treated with GRR crude extract and the control group increased with time. All the rats had drastically gained weight during the experiment. There was a significant increase of body weight in G4 and G2 as compared to the control group (G1) (*p* < 0.05).

Table 1 shows the final organ weight ratio (organ weight/final animal weight) of the animals treated and untreated with GRR crude extract. There were no significant differences in the weight ratio of liver and kidney from all the groups treated with GRR crude extract compared to the control (G5) (*p* > 0.05). There were no significant differences in colon weight ratio between G1, G2, and G3 (*p* > 0.05). There were significant differences in the heart, lung, and spleen weight ratio between G2, G3, and G4 (*p* < 0.05). A significant increase of colon weight ratio was noted in G4 as compared to other groups (G1, G2, and G3) (*p* < 0.05).

Hematological values of the animals treated and untreated with GRR crude extract are shown in Table 2. There were no significant differences (*p* > 0.05) in the values for red blood cells (RBC), haemoglobin (HGB), hematocrit (HCT), mean corpuscular volume (MCV), mean corpuscular haemoglobin (MCH), and mean corpuscular haemoglobin concentration (MCHC) between all the groups treated with GRR crude extract compared to the control group (G5). There were significant differences in the value of white blood cells (WBC) in G1 and G2 compared to G3, G4, and G5 (*p* < 0.05).

### 3.3. Effect of Germinated Rough Rice Crude Extract on the Incidence of Aberrant Crypt Foci

The effect of GRR crude extract on AOM-induced aberrant crypt foci (ACF) development in rats is summarized in Table 3. A significant reduction in the total number of ACF, AC, and multicrypt of ACF per colon in all the groups treated with GRR crude extract compared to the control group (G1) was noted (*p* < 0.05). Reduction in the total number of ACF and multicrypt of ACF with increase in the percentage of GRR crude extract was observed but yet insignificant (*p* > 0.05). The lowest total number of ACF was noted in G4.

### 3.4. Histological Classification of ACF

**Figure 3:**
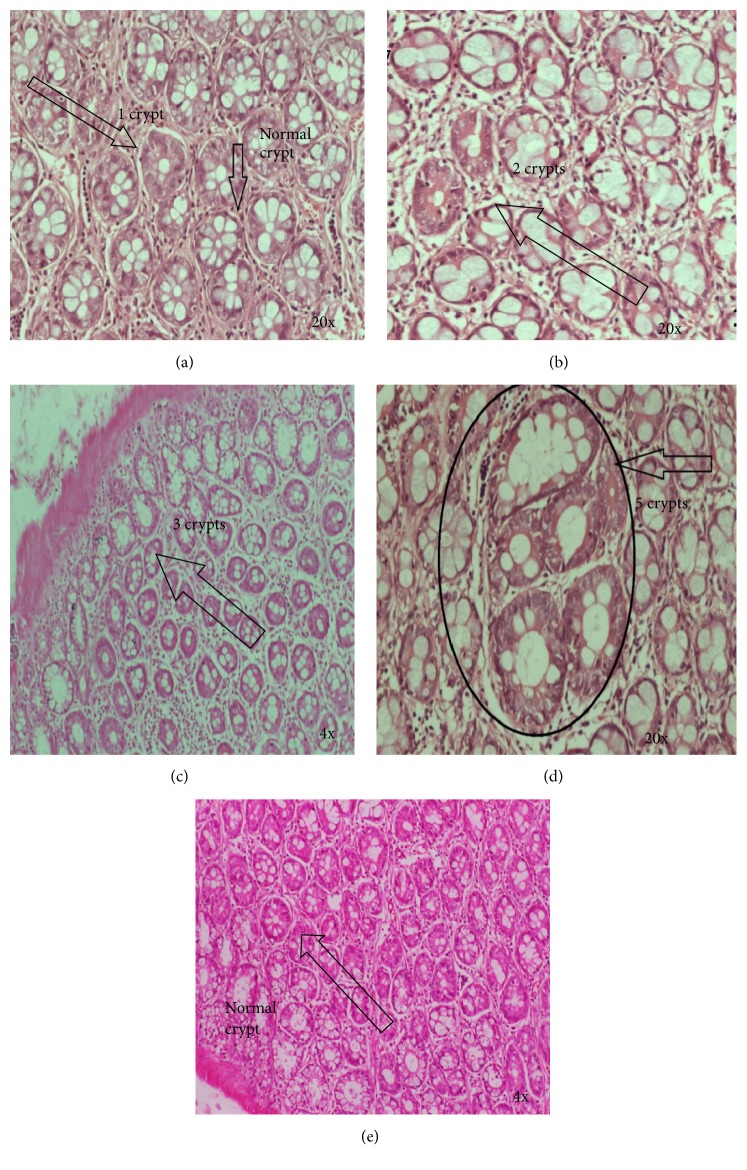
Histology of aberrant crypt foci with different number of crypts.

The morphology of ACF at 8 weeks after administration with GRR crude extract was evaluated. ACF were classified based on the established histological criteria.  Figure 3 shows the histology of different size of ACF. The animals in G1 had the highest amount of total number of ACF.

The animals in G4 had the lowest number of 1, 2, 3, and 4 crypts. There is a lack of goblet cell differentiation, thickening of the epithelium, and nuclear elongation and stratification, indicative of moderate to severe dysplasia (Figure 3(b)). A large ACF with five crypts shows a wide range of morphological characteristics from hyperplasia to severe dysplasia (Figure 3(d)).

### 3.5. Expression of *β*-Catenin

**Figure 4:**
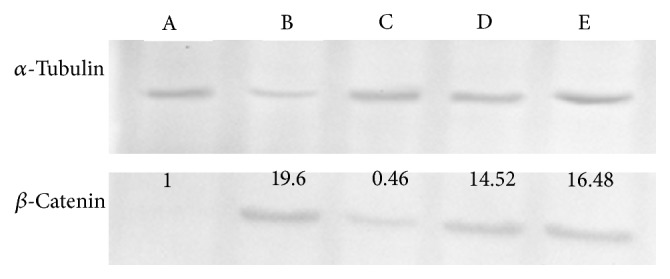
Western blot analysis of the expression of *β*-catenin in rats induced with AOM treated with different dose of GRR crude extract. Fold change was normalized against *α*-tubulin and compared to the control. Lane A: normal group (G5). Lane B: azoxymethane alone (G1). Lane C: AOM + 2000 mg GRR crude extract/kg body weight (G4). Lane D: AOM + 1000 mg GRR crude extract/kg body weight (G3). Lane E: AOM + 500 mg GRR crude extract/kg body weight (G2).

Western blot analysis of the expression of *β*-catenin is shown in Figure 4. Untreated group (G1) showed the highest level of *β*-catenin expression which was verified by quantification of the intensity of the protein bands by densitometry analysis. The expressions of *β*-catenin were decreased by increasing the administration of GRR crude extract. The lowest level of *β*-catenin expression was from G4 (*p* < 0.05).

## 4. Discussion

### 4.1. Cytotoxic Properties of Germinated Rough Rice Crude Extract

The consumption of whole grains results in a reduced risk of emerging cancer diseases. This could be attributed to the presence of natural antioxidants such as *γ*-aminobutyric acid, tocopherols, tocotrienols, and phenolic compounds (polyphenols) [24]. In the present study, ethanol was selected as the extraction medium because it is less toxic and much safer than methanol [25]. Nevertheless, not much study has been conducted on GRR especially on its anticancer properties. Therefore, not much discussion can be made except the one found from the present study. In the previous study, the concentration of total phenolics in the grain has been positively related with the antioxidant activity [5].

Germination is believed to increase the level of bioactive compounds of rough rice such as phenolic compounds, p-coumaric acid [26, 27], chlorogenic acid, hydroxybenzoic acid [26], vanillic acid, syringic acid, caffeic acid [26, 27], oryzanol, GABA, and vitamin E, which have been shown to have antioxidant activity [28].

The GRR crude extract was cytotoxic to the HT29 cells. According to the National Cancer Institute, USA, the agent that shows cytotoxicity below 100 *μ*g/mL has the potential to be developed as an anticancer agent. Previous studies have demonstrated that polyphenolic compounds have been associated with the scavenging of free radicals and the enhancement of immune systems, which can reduce the risk of developing cancer [29], can affect the process of carcinogenesis by counteracting the occurrence of oxidative stress, and can prevent the inception and development of cancer [30].

### 4.2. Chemopreventive Properties of Germinated Rough Rice Crude Extract in Rats Induced with Azoxymethane

Colon carcinogenesis models using AOM with putative preneoplastic ACF as an end point marker lesion have been used to assess the influence of modulatory factors [31, 32].

The results demonstrated that administration of GRR crude extract suppressed the number of ACF and multicrypt of ACF per colon. The chemopreventive effects of GRR crude extract were dose-dependent.

ACF have been classified as hyperplastic (nondysplastic) or dysplastic based primarily on the morphological characteristic and the final harbouring of *β-catenin* and/or* Apc* mutations. So, it is demonstrating a mutation range similar to previous study in colon cancers [13, 33, 34]. Treatment with GRR crude extract reduced the dysplastic changes, which suggests that the extract is able to inhibit the progression of colon cancer in the early stage.

Many studies emphasized the importance of large ACF composed of four or more aberrant crypts [16]. Critical changes in ACF which are essential for further development to dysplastic ACF may also be presented or occurred even in small lesions. The severity of the degree of luminal alteration may relate to the degree of dysplasia present in particular aberrant crypt [35].

A central pathway of cancer inhibition by GRR crude extract is not deliberated yet. Nevertheless, other studies on the other variety of rice and germinated brown rice indicated that they reduced the rate of cellular proliferation both in vivo and in vitro [10, 16, 36].

It has been reported that inositol hexaphosphate (IP6) which is a chemical found in bran layer or bran oil of rice, wheat, and other high-fiber foods reduced the carcinogen-induced large bowel cancer and inhibited growth of transplanted tumors. However, the mechanism by which IP6 exerts chemopreventive activity is not completely understood. We believe that the chemopreventive properties of GRR crude extract are as a result of the nutritional components such as phytic acid (IP6), ferulic acid, inositol, and dietary fiber which were increased in the content after the process of germination [36]. Ferulic acid, which is a phenolic phytochemical extracted from bran rice, inhibited the growth of colonic ACF and suppressed the progression of preneoplastic to malignant neoplasia. The suppression of metabolic activation and enhancement of detoxification have been postulated to be the mechanisms for chemopreventive action of ferulic acid [37].

Treatment with GRR crude extract reduced the expression of *β-catenin*. Mutations in *β-catenin* gene cause cytosolic growth of *β*-catenin and, consequently, the increase of transcriptional activity of the *β*-catenin-T cell-factor/lymphoid-enhancer-factor complex. Since GRR crude extract has shown chemopreventive properties, it is recommended to examine its anticancer properties for future study. It is also recommended to determine the mechanisms of action of how GRR crude extract inhibits the progression of colon cancer. This process seems to play an essential role in the development of most colorectal carcinomas.

## Figures and Tables

**Table 1:** Organ weight ratios (organ weight/final animal weight) after the chemopreventive properties of germinated rough rice (GRR) crude extract.

Group	Treatment	Kidney	Liver	Heart	Lung	Spleen	Colon
1	AOM alone	0.71 ± 0.06^b^	2.45 ± 0.1^b^	0.34 ± 0.03^c^	0.64 ± 0.06^d^	0.19 ± 0.01^c^	0.6 ± 0.06^b^
2	AOM + 500 mg/kg	0.7 ± 0.06^b^	2.42 ± 0.32^b^	0.34 ± 0.06^c^	0.85 ± 0.01^c^	0.16 ± 0.02^d^	0.56 ± 0.08^b^
3	AOM + 1000 mg/kg	0.79 ± 0.07^b^	2.48 ± 0.14^b^	0.38 ± 0.05^bc^	0.97 ± 0.09^bc^	0.19 ± 0.02^cd^	0.57 ± 0.06^b^
4	AOM + 2000 mg/kg	0.8 ± 0.1^b^	2.36 ± 0.29^b^	0.49 ± 0.07^a^	1.2 ± 0.15^a^	0.26 ± 0.02^b^	0.67 ± 0.1^ab^
5	Normal	0.95 ± 0.09^a^	3.09 ± 0.33^a^	0.41 ± 0.01^a^	1.06 ± 0.11^ab^	0.30 ± 0.02^b^	0.74 ± 0.1^a^

Each value represents the mean ± SEM (*n* = 6). Values in the same column with different letter superscripts differ significantly as determined by Tukey's multiple range test (*p* < 0.05).

**Table 2:** Haematological value after the chemopreventive properties of germinated rough rice (GRR) crude extract.

Treatment	WBC (×10^3^)	RBC (×10^6^)	HGB (g/dL)	HCT (%)	MCV (pg)	MCH (fL)	MCHC (g/dL)
AOM alone	14.74 ± 1.2^a^	8.52 ± 0.16^a^	15.34 ± 0.59^a^	46.8 ± 1.88^a^	55.74 ± 0.84^a^	18 ± 0.57^a^	32.8 ± 0.41^a^
AOM + 500 mg/kg	14.7 ± 2.75^ab^	8.396 ± 0.12^a^	15.06 ± 0.16^a^	47.28 ± 0.46^a^	56.26 ± 0.72^a^	17.84 ± 0.2^a^	31.9 ± 0.27^a^
AOM + 1000 mg/kg	10.76 ± 0.67^c^	8.496 ± 0.56^a^	15.5 ± 0.52^a^	47.64 ± 1.9^a^	56.18 ± 1.66^a^	18.28 ± 0.85^a^	32.56 ± 0.70^a^
AOM + 2000 mg/kg	10.54 ± 1.02^c^	9.03 ± 0.38^a^	15.44 ± 0.97^a^	49.4 ± 2.65^a^	54.52 ± 0.95^a^	17.3 ± 0.63^a^	31.64 ± 0.53^a^
Normal	11.56 ± 1.80^bc^	8.76 ± 0.41^a^	15.64 ± 0.7^a^	49.18 ± 3.2^a^	55.64 ± 0.81^a^	17.82 ± 0.13^a^	31.84 ± 0.92^a^

Each value represents the mean ± SEM (*n* = 6). Values in the same column with different letter superscripts differ significantly as determined by Tukey's multiple range test (*p* < 0.05).

**Table 3:** The effect of germination of rough rice (GRR) crude extract on the incident of ACF in rat colon induced with azoxymethane (AOM).

Group	Treatment	1 crypt	2 crypts	3 crypts	4 or more crypts	Total number of ACF/colon
1	AOM alone	15.67 ± 2.08^A,a^	11.67 ± 3.51^AB,a^	8.33 ± 1.53^B,a^	11 ± 1.73^AB,a^	46.67 ± 2.52^a^
2	AOM + 500 mg/kg	13 ± 1^A,ab^	7.67 ± 1.15^B,a^	8 ± 2.65^B,ab^	10 ± 1.73^AB,ab^	38.67 ± 2.52^b^
3	AOM + 1000 mg/kg	8.67 ± 1.53^A,bc^	8 ± 2^A,a^	5.67 ± 2.08^A,a^	7.67 ± 1.15^A,ab^	30 ± 1.73^c^
4	AOM + 2000 mg/kg	8 ± 2^A,c^	7 ± 1.73^A,a^	5.67 ± 0.58^A,a^	5.67 ± 2.89^A,b^	26.33 ± 2.08^c^
5	Normal	0	0	0	0	0

Each value is expressed as mean ± SD (*n* = 6). Different capital letters show significant difference in each row and different small letters show significant difference in each column as determined by Tukey's multiple range test (*p* < 0.05).
